# Supplementary material for: Audiogenic Seizures and Social Deficits: No Aggravation Found in Krushinsky–Molodkina Rats
Source: Biomedicines. 2023 Sep 18;11(9):2566. doi: 10.3390/biomedicines11092566 (PMC10526393; doi:10.3390/biomedicines11092566)
Supplement: Supplementary file 1 [file biomedicines-11-02566-s001.zip › biomedicines-2531201-supplementary.pdf]

Table S1A. Behavior in the "elevated plus maze" test, between groups comparisons, the 1st (pre-provocation) trial

	**KM rats (mean±Std. er. of mean)**	**Wistar rats (mean±Std. er. of mean)**	**p-value**
Path length, m	7,2±0,6	19,6±1,7	0,00002
Percentage of time spent in open arms	6,2±3,4	16,3±2,4	0,004
Percentage of time spent in closed arms	66,8±4,6	76,5±3,3	0,11
Closed/open-arms entry ratio	3,3±0,8	3,3±0,8	0,76
Falls	0,1±0,1	0,1±0,1	0,94
Number of rearings	12,3±1,2	20,4±1,9	0,006
Number of short grooming	10,0±1,2	2,8±0,8	0,00003
Number of hole body grooming	1,9±0,3	1,7±0,4	0,37
Number of freezing episodes	20,5±2,0	10,6±2,1	0,001
Total freezing time, sec	212,2±28,9	84,5±26,6	0,0003
Stereotypy (head waiving)	1,5±0,3	0,6±0,3	0,05

Statistical significance is calculated according to Mann-Whitney U-test. Significantly differing parameters are underlined. Data are represented as the mean ± SEM

Table S1B. Behavior in the “elevated plus maze” test, between-groups comparisons, the 2nd (post-provocation) trial

	**KM rats (mean±Std. er. of mean)**	**Wistar rats (mean±Std. er. of mean)**	**p-value**
Path length, m	15,9±1,2	15,7±1,5	0,69
Percentage of time spent in open arms	1,7±0,6	13,2±4,8	0,08
Percentage of time spent in closed arms	78,1±4,9	53,8±7	0,007
Closed/open-arms entry ratio	4,1±0,9	3,3±1,2	0,34
Falls	0±0	0±0	
Number of rearings	9,9±0,7	20,3±1,7	0,000009
Number of short grooming	7,7±0,9	2,3±0,6	0,00003
Number of whole-body grooming	1,8±0,2	1,3±0,2	0,17
Number of freezing episodes	13,9±2,6	19±2,4	0,10
Total freezing time, sec	150,5±29,9	137±23,3	0,89
Stereotypy (head waiving)	0,3±0,2	0,1±0,1	0,36

Statistical significance is calculated according to Mann-Whitney U-test. Significantly differing parameters are underlined. Data are represented as the mean ± SEM

Table S1C. Locomotor behavior in the “elevated plus maze” test, within-groups comparisons

**KM rats, sound provocation subgroup**	**Pre-provocation**	**Post-provocation**	**p-value**
Path length, m	7,3±0,9	16,5±1,9	0,001
Number of freezing episodes	19,0±3,7	9,4±2,0	0,04
Total freezing time, sec	217,1±46,5	124,7±43,6	0,10

**KM rats, sham subgroup**	**Before sham provocation**	**After sham provocation**	**p-value**
Path length, m	7,1±0,9	15,2±1,7	0,003
Number of freezing episodes	22,1±1,8	18,5±4,3	0,14
Total freezing time, sec	207,2±38,8	176,3±41,7	0,57

**Wistar rats, sound provocation subgroup**	**Pre-provocation**	**Post-provocation**	**p-value**
Path length, m	17,1±2,9	14,4±2,5	0,44
Number of freezing episodes	12,9±3,3	19,3±3,4	0,23
Total freezing time, sec	124,8±48,3	142,5±36,4	0,50

**Wistar rats, sham provocation subgroup**	**Before sham provocation**	**After sham provocation**	**p-value**
Path length, m	22,0±1,7	17±1,8	0,07
Number of freezing episodes	8,4±2,4	18,8±3,5	0,009
Total freezing time, sec	44,2±15,4	131,4±31,5	0,009

Statistical significance is calculated according to Wilcoxon matched pair test. Significantly differing parameters are written in color. Data are represented as the mean ± SEM

Table S1D. Behavior in the "elevated plus maze" test, the 2nd (post-provocation) trial. Comparison between KM subgroups: AGS-experienced and sham-stressed rats

	**KM rats, sound provocation (mean±Std. er. of mean)**	**KM rats, sham provocation (mean±Std. er. of mean)**	**p-value**
Path length, m	16,5±1,9	15,3±1,7	1
Percentage of time spent in open arms	2,2±1,0	1,2±0,5	0,64
Percentage of time spent in closed arms	76,6±7,4	79,6±6,8	1
<u>*Closed/open-arms entry ratio*</u>	<u>5,9±1,1</u>	<u>2,4±1,0</u>	<u>0,04</u>
Falls	0±0	0±0	
Number of rearings	9,9±0,4	10,0±1,3	1
Number of short grooming	8,8±1,6	6,6±0,9	1
Number of whole-body grooming	1,9±0,4	1,7±0,4	1
Number of freezing episodes	9,4±2,0	18,5±4,3	0,08
Total freezing time, sec	124,7±43,6	176,3±41,7	0,76
Stereotypy (head waiving)	0,3±0,3	0,4±0,2	1

Statistical significance is calculated according to Mann-Whitney U-test, with Bonferroni corrections. Significantly differing parameters are underlined. Data are represented as the mean ± SEM

Table S2. Two-choice social preference test, between-groups comparisons

	КМ rats (mean±Std. er. of mean)	Wistar rats (mean±Std. er. of mean)	p-value
**1st session, Social preference test**			
Time spent in stimulus rat zone, sec	218,1±92,6	317,1±75,1	0,69
Time spent in empty cage zone, sec	342,0±99,2	245,8±73,0	0,70
Number of contacts with stimulus rat	4,1±1,7	10,4±3,6	0,20
Number of contacts with empty cage	2,3±0,7	1,6±0,8	0,97
Number of short contacts with stimulus rat	4,1±1,7	9,5±3,4	0,28
Number of long contacts with stimulus rat	0±0	0,9±0,5	0,23
Total time of contact with stimulus rat, sec	2,7±1,3	12,0±4,0	0,04
Total time of contact with empty cage, sec	1,9±0,8	0,1±0,1	0,926
Total freezing time, sec	272,0±48,7	97,1±21,7	0,02
**2nd session, Social novelty test**			
Time spent in familiar stimulus rat zone, sec	284,0±105,7	299,0±78,5	0,97
Time spent in unfamiliar stimulus rat zone, sec	261,9±102,6	277,4±79,7	0,99
Number of contacts with familiar stimulus rat	2,0±0,9	2,0±0,5	1
Number of contacts with unfamiliar stimulus rat	3,3±1,6	5,0±1,4	0,51
Number of short contacts with familiar stimulus rat	2±0,9	2±0,5	0,77
Number of long contacts with unfamiliar stimulus rat	0±0	0±0	
Number of short contacts with familiar stimulus rat	3,1±1,5	4,4±1,2	0,39
Number of long contacts with unfamiliar stimulus	0,1±0,1	0,6±0,4	0,89

rat			
Total time of contact with familiar stimulus rat, sec	1,8±0,8	2,7±0,8	0,86
Total time of contact with unfamiliar stimulus rat, sec	5,4±3,0	10,7±4,0	0,08
<u>*Total freezing time, sec*</u>	<u>337,6±32,3</u>	<u>126,2±21,2</u>	<u>0,00</u>

Statistical significance is calculated according to Mann-Whitney U-test. Significantly differing parameters are underlined. Data are represented as the mean ± SEM

Table S3A. Socially enriched open field test, the 1st trial (pre-provocation), between-groups comparisons

	KM rats (mean±Std. er. of mean)	Wistar rats (mean±Std. er. of mean)	p-value
Path length, m	18,3±2,8	45,8±4,9	0,0002
% of time in stimulus rat zone	54,9±5,9	43,4±2,6	0,08
% path in the stimulus rat zone	22,6±3,8	37,8±5,5	0,02
Number of rearings	39,8±5,1	43,3±3,4	0,76
Number of short grooming	17,8±2,0	5,6±0,5	0,000009
Number of long grooming	1,3±0,4	1,2±0,3	0,98
Number of short contacts	5,1±1,0	8,9±1,7	0,08
Number of long contacts	4,2±1,0	9,7±1,5	0,007
Defecation	1,3±0,5	1,4±0,5	0,98
Number of freezing episodes	38,1±6,2	24,0±3,8	0,03
Total freezing time, sec	286,2±58,6	224,7±50,9	0,28
Stereotypy (head waiving)	0,3±0,1	0,3±0,1	0,945

Statistical significance is calculated according to Mann-Whitney U-test. Significantly differing parameters are underlined. Data are represented as the mean ± SEM

Table S3B. Socially enriched open field test, the locomotor parameters (within-groups comparisons)

KM rats, sound provocation	Pre-provocation	Post-provocation	p-value
Path length, m	21,7±3,7	38,6±6,8	0,10
Number of freezing episodes	42,3±11,4	25,5±4,4	0,28
Total freezing time, sec	319,2±112,5	355,6±104,1	0,85

KM rats, sham provocation	Before sham provocation	After sham provocation	p-value
Path length, m	15,0±3,9	41,0±6,4	0,008
Number of freezing episodes	33,8±5,5	30,9±6,8	1
Total freezing time, sec	253,2±45,2	360,0±89,3	0,85

Wistar rats, sound provocation	Pre-provocation	Post-provocation	p-value
Path length, m	46,1±5,3	51,4±8,1	0,50
Number of freezing episodes	20,8±4,3	25,6±7,4	0,79
Total freezing time, sec	212±78,6	224,4±79,7	0,95

Wistar rats, sham provocation	Before sham provocation	After sham provocation	p-value
Path length, m	45,6±8,7	65±6,1	0,105
Number of freezing episodes	27,3±6,5	19±3,4	0,505
Total freezing time, sec	237,4±69,7	138,5±29,7	0,442

Statistical significance is calculated according to Wilcoxon matched pair test. Significantly differing parameters are written in color. Data are represented as the mean ± SEM

Table S3C. Socially enriched open field, between subgroups comparisons: Comparison between KM subgroups: AGS-experienced and sham-stressed rats

	**KM sound provocation (mean±SEM)**	**KM sham provocation (mean±SEM.)**	**p-value**
Path length, m	38,6±6,8	41±6,4	1
% path in the stimulus rat zone	9,9±2,5	18,2±3,8	0,13
Number of rearings	21,5±6,3	29,4±6,6	0,78
Number of short grooming	9,1±2,5	14,6±2,6	0,05
Number of long grooming	1,4±0,4	0,9±0,3	0,76
Number of freezing episodes	25,5±4,4	30,9±6,8	1
Number of short contacts	2,6±1,1	3,9±1,5	1
Number of long contacts	2,1±0,6	3,1±1,1	1
Defecation	0,1±0,1	0,5±0,2	0,47
Stereotypy (head waiving)	0,3±0,2	0,3±0,3	1

Statistical significance is calculated according to Mann-Whitney U-test, with Bonferroni corrections. Significantly differing parameters are written in color. Data are represented as the mean ± SEM

Table S3D. Socially enriched open field test, the 2nd trial (post-provocation), between groups comparisons

	KM rats (mean±Std. er. of mean)	***Wistar rats (mean±Std. er. of mean)***	**p-value**
Path length, m	39,8±4,5	58,2±5,2	0,02
% of time in stimulus rat zone	10,6±1,7	21,3±2,7	0,006
% path in the stimulus rat zone	14,1±2,4	21,3±2,4	0,03
Number of rearings	25,4±4,5	44,3±5,3	0,02
Number of short grooming	11,9±1,9	6,1±1,3	0,002
Number of long grooming	1,1±0,2	0,9±0,3	0,42
Number of freezing episodes	28,2±4,0	22,3±4,0	0,30
Total freezing time, sec	357,8±66,3	181,5±42,6	0,02
Number of short contacts	3,3±0,9	8,6±1,5	0,003
Number of long contacts	2,6±0,6	10,7±1,3	0,00001
Stereotypy (head waiving)	0,3±0,1	0±0	0,38

Statistical significance is calculated according to Mann-Whitney U-test. Significantly differing parameters are underlined. Data are represented as the mean ± SEM

Table S4A Three-chambered social preference test, the 1st trial (pre-provocation), between-groups comparisons

	KM rats (mean±Std. er. of mean)	*Wistar rats (mean±Std. er. of mean)*	*p-value*
Path length, m	15,8±3	22,2±2,4	0,09
Path length in stimulus rat compartment, m	4,2±1,3	10,5±1,2	0,0004
Path length in the empty cage compartment, m	5,4±1	6±0,9	0,56
Path length in the intermediate compartment, m	6±1,3	5,7±0,7	0,98
Time spent in stimulus rat compartment, sec	106,5±21,1	187,8±19,5	0,02
Time spent in the empty cage compartment, sec	317,6±47	173,2±16,8	0,009
Time spent in the intermediate compartment, sec	175,6±33,3	239,1±25,4	0,18
Number of rearings	9,1±1,9	14±1,8	0,09
Number of short grooming	3,8±0,9	5±0,9	0,39
Number of long grooming	1,3±0,6	1±0,2	0,59
Number of contacts with stimulus rat	1,5±0,6	6,3±0,8	0,00003
Number of freezing episodes	17,7±3,8	9,1±1,8	0,06
Total freezing time, sec	188,7±46,7	56,9±13,7	0,04
Stereotypy (head waiving)	1,4±0,7	0,8±0,4	0,8

Statistical significance is calculated according to Mann-Whitney U-test. Significantly differing parameters are underlined. Data are represented as the mean ± SEM

Table S4B. Three-chambered social preference test, within-groups comparisons of locomotor parameters

KM rats, sound provocation subgroup	Pre-provocation	Post-provocation	p-value
Path length, m	15,7±5,4	25,3±5,5	0,31
Number of freezing episodes	17,3±4,6	11,3±6,8	0,18
Average freezing time, sec	9,2±2,2	5,5±0,7	0,39
Total freezing time, sec	195,8±73,9	82,6±56,3	0,13

KM rats, sham provocation subgroup	Before sham provocation	After sham provocation	p-value
Path length, m	16,0±3,2	27,5±3,3	0,03
Number of freezing episodes	18,0±6,4	8,0±4,1	0,12
Total freezing time, sec	181,6±64,1	45,2±23,5	0,05

Wistar rats, sound provocation subgroup	Pre-provocation	Post-provocation	p-value
Path length, m	21,5±3,8	29,2±5,3	0,27
Number of freezing episodes	9,3±2,5	5,6±1,9	0,16
Average freezing time, sec	6,1±0,4	10,3±5,1	0,50
Total freezing time, sec	59,6±19,6	51,3±20,1	0,44

Wistar rats, sham provocation subgroup	Before sham provocation	After sham provocation	p-value
Path length, m	23±3,3	30,2±2,2	0,16
Number of freezing episodes	8,9±2,8	4,6±0,9	0,38
Average freezing time, sec	5,2±0,5	8,2±1,8	0,23
Total freezing time, sec	54,3±20,4	43,2±14,1	0,79

Statistical significance is calculated according to Wilcoxon matched pair test. Significantly differing parameters are written in color. Data are represented as the mean ± SEM

Table S4C. Three-chambered social preference test, 2nd trial (post-provocation). Comparison between KM subgroups: AGS-experienced and sham-stressed rats

	KM* sound provocation *(mean±SEM)	***KM* sham provocation *(mean±SEM)***	***p-value***
Path length, m	25,3±5,5	27,5±3,3	1
Path length in stimulus rat compartment, m	8,0±2,8	7,9±1,2	1
Path length in the empty cage compartment, m	10,7±1,1	10,0±2,0	1
Path length in the intermediate compartment, m	6,0±2,2	9,7±1,7	1
Number of rearings	2,5±1,1	2,2±0,9	1
Number of short grooming	3,8±1,0	2,4±1,5	0,84
Number of long grooming	0,3±0,3	0,2±0,2	1
Number of short contacts with stimulus rat	4,5±0,8	5,8±1,2	1
Number of long contacts with stimulus rat	2,7±0,7	3,2±0,5	0,65
Time spent in stimulus rat compartment, sec	121,3±41,7	216,2±23,2	0,35
Time spent in the empty cage compartment, sec	291,4±42,2	207,7±28,3	0,50
Time spent in the intermediate compartment, sec	187,3±19,3	176,1±30,8	1
Number of freezing episodes	11,3±6,8	8,0±4,1	1
Total freezing time, sec	82,6±56,3	45,2±23,5	1
Stereotypy (head waiving)	0±0	0±0	

Statistical significance is calculated according to Mann-Whitney U-test, with Bonferroni corrections. Significantly differing parameters are written in color. Data are represented as the mean ± SEM

Table S4D. Three-chambered social preference, the 2nd trial (post-provocation), between-groups comparisons

	KM rats (mean±Std. er. of mean)	*Wistar rats (mean±Std. er. of mean)*	*p-value*
Path length, m	26,3±3,2	29,7±2,8	0,57
Path length in stimulus rat compartment, m	7,9±1,5	9,8±1,1	0,45
Path length in the empty cage compartment, m	10,4±1,1	12,5±1,6	0,34
Path length in the intermediate compartment, m	8,0±1,5	7,4±0,9	0,90
Time spent in stimulus rat compartment, sec	164,4±28,2	266,1±26,5	0,01
Time spent in the empty cage compartment, sec	253,4±28,4	187±20,2	0,09
Time spent in the intermediate compartment, sec	182,2±16,7	146,9±18,8	0,21
Number of short grooming	2,4±0,7	9,1±1,3	0,0002
Number of long grooming	0,3±0,2	1,4±0,2	0,003
Number of short contacts with stimulus rat	3,2±0,9	3,7±1,0	0,90
Number of long contacts with stimulus rat	2,9±0,4	4,0±0,5	0,16
Number of freezing episodes	9,8±4,0	5,1±1,0	0,79
Total freezing time, sec	65,6±31,6	47,2±11,9	0,54
Stereotypy (head waiving)	0±0	0±0	

According to Mann-Whitney U-test. Significantly differing parameters are underlined. Data are represented as the mean ± SEM

Table S5A. Three-chambered social novelty, the 1st trial (pre-provocation), between-groups comparisons

	KM rats (mean±Std. er. of mean)	*Wistar rats (mean±Std. er. of mean)*	*p-value*
Path length, m	4,0±0,8	12,6±1,7	0,00004
Path length in familiar stimulus rat compartment, m	0,6±0,3	3,6±0,6	0,0003
Path length in the unfamiliar stimulus rat compartment, m	2,0±0,5	5,3±0,8	0,002
Path length in the intermediate compartment, m	1,6±0,5	3,6±0,6	0,02
Time spent in familiar stimulus rat compartment, sec	61,0±24,4	196,3±31,7	0,002
Time spent in the unfamiliar stimulus rat compartment, sec	271,1±51,4	272,0±26,6	0,69
Time spent in the intermediate compartment, sec	271,4±50,9	131,7±15,9	0,0003
Number of rearings	1,4±0,5	8,6±1,3	0,00002
Number of short grooming	1,8±0,7	4,1±1,0	0,06
Number of long grooming	0±0	1,4±0,2	0,0001
Number of contacts with familiar stimulus rat	0,3±0,1	2,7±0,3	0,000001
Number of contacts with unfamiliar stimulus rat	0,8±0,3	4,1±0,7	0,001
Number of freezing episodes	19,3±3,2	18,2±2,4	0,66
Total freezing time, sec	467,6±20,1	135,2±27,1	0,00
Stereotypy (head waiving)	0,9±0,4	0,6±0,2	0,83

Statistical significance is calculated according to Mann-Whitney U-test. Significantly differing parameters are underlined. Data are represented as the mean ± SEM

Table S5B. Three-chambered social novelty test, within-groups comparisons (the locomotor parameters between the two trials)

KM rats, sound provocation subgroup	Pre-provocation	Post-provocation	p-value
Path length, m	3,1±0,7	14,8±3,4	0,009
Number of freezing episodes	18,0±5,9	16,3±2,6	0,81
Total freezing time, sec	480,5±27,5	196,8±55,6	0,002

KM rats, sham provocation subgroup	Before sham provocation	After sham provocation	p-value
Path length, m	4,9±1,5	7,0±2,8	0,66
Number of freezing episodes	20,5±3,0	18,4±2,1	0,24
Total freezing time, sec	454,6±31	319,5±79,9	0,32

Wistar rats, sound provocation	Pre-provocation	Post-provocation	p-value
Path length, m	14,1±2,7	18,3±3,1	0,27
Number of freezing episodes	15,8±3,8	10,4±1,3	0,27
Total freezing time, sec	117,1±45	64,2±10,7	0,44

Wistar rats, sham provocation	Before sham provocation	After sham provocation	p-value
Path length, m	11,0±2,0	19,4±1,6	0,01
Number of freezing episodes	20,6±2,9	11,6±2,0	0,021
Total freezing time, sec	153,2±31,9	73,5±14,1	0,06

Statistical significance is calculated according to Wilcoxon matched pair test. Significantly differing parameters are written in color. Data are represented as the mean ± SEM

Table S5C. Three-chambered social novelty test, 2nd trial (post-provocation), between KM subgroups comparison

	KM **sound provocation *(mean±SEM)***	***KM*** **sham provocation *(mean±SEM)***	***p-value***
Path length, m	14,8±3,4	7,0±2,8	0,50
Path length in stimulus rat compartment, m	4,6±0,9	2,8±1,4	065
Path length in the empty cage compartment, m	4,9±1,3	1,5±1,1	0,35
Path length in the intermediate compartment, m	5,2±1,6	2,7±0,5	0,85
Time spent in stimulus rat compartment, sec	282,4±64,5	206,8±68,7	1
Time spent in the empty cage compartment, sec	174,9±41,8	96,5±57,7	0,85
Time spent in the intermediate compartment, sec	142,7±24,1	296,8±69,4	0,50
Number of rearings	7,5±2,2	10,2±3,2	1
Number of short grooming	2,7±0,9	3,6±1,3	1
Number of long grooming	2,0±1,2	0,2±0,2	0,35
Number of short contacts with familiar stimulus rat	2,2±1,2	2,0±0,6	1
Number of long contacts with familiar stimulus rat	0,5±0,3	0,2±0,2	1
Number of short contacts with unfamiliar stimulus rat	3,7±1,3	0,4±0,2	0,06
Number of long contacts with unfamiliar stimulus rat	2,2±0,7	0,2±0,2	0,06
Number of freezing episodes	16,3±2,6	18,4±2,1	1
Total freezing time, sec	196,8±55,6	319,5±79,9	0,50
Stereotypy (head waiving)	0,5±0,2	0,2±0,2	0,85

Statistical significance is calculated according to Mann-Whitney U-test, with Bonferroni corrections. Significantly differing parameters are written in color. Data are represented as the mean ± SEM

Table S5D. Three-chambered social novelty test, the 2nd trial (post-provocation), between-groups comparisons

	KM rats (mean±Std. er. of mean)	*Wistar rats (mean±Std. er. of mean)*	*p-value*
Path length, m	11,3±2,5	18,9±1,7	0,02
Path length in stimulus rat compartment, m	3,8±0,8	6,7±0,8	0,01
Path length in the empty cage compartment, m	3,4±1,0	7,3±1,2	0,09
Path length in the intermediate compartment, m	4,1±0,9	4,9±0,8	0,45
Time spent in stimulus rat compartment, sec	248,0±46,2	209,4±20,7	0,68
Time spent in the empty cage compartment, sec	139,3±35,1	217,4±22,5	0,16
Time spent in the intermediate compartment, sec	212,8±40,3	173,2±19,6	0,64
Number of rearings	8,7±1,8	14,4±1,8	0,08
Number of short grooming	3,1±0,7	4,9±1,0	0,25
Number of long grooming	1,2±0,7	0,6±0,2	0,94
Number of short contacts with familiar stimulus rat	2,1±0,7	3,1±0,6	0,12
Number of long contacts with familiar stimulus rat	0,4±0,2	2,8±0,4	0,00007
Number of short contacts with unfamiliar stimulus rat	2,2±0,9	4,6±0,7	0,02
Number of long contacts with unfamiliar stimulus rat	1,3±0,5	2,5±0,2	0,02
Number of freezing episodes	17,3±1,6	11,0±1,1	0,008
Total freezing time, sec	252,6±48,8	68,8±8,6	0,001

Stereotypy (head waiving)	0,4±0,2	0±0	0,12

Statistical significance is calculated according to Mann-Whitney U-test. Significantly differing parameters are underlined. Data are represented as the mean ± SEM

Table S6. Two-objects/novel object recognition test, between-groups comparisons

**the 1st session, 2 new objects**	**КМ rats *(mean±SEM)***	**Wistar rats, *(mean±SEM)***	**p-value**
Path length, m	29,1±2,8	30,5±3	0,65
Number of rearings	22,4±1,8	24,2±3,3	0,85
Number of short grooming	5,0±0,7	3,5±0,4	0,07
Number of long grooming	2,5±0,5	2,1±0,5	0,40
Mean number of contacts with objects	6,95±1,1	7,9±0,6	0,34
Mean number of active contacts with objects	2,6±0,7	0,4±0,2	0,12
Number of freezing episodes	6,9±1,5	10,4±2,7	0,37
Total freezing time, sec	38,5±9,2	77,5±25,4	0,24
Stereotypy (head waiving)	0,8±0,3	0,9±0,3	0,93

Statistical significance is calculated according to Mann-Whitney U-test. Significantly differing parameters are underlined. Data are represented as the mean ± SEM

**the 2nd session, the "old" and "new" objects**	**КМ rats *(mean±SEM)***	**Wistar rats, *(mean±SEM)***	**p-value**
Path length, m	15,0±2,2	12,4±62,2	0,35
Number of rearings	7,2±1,4	6,4±1,8	0,41
Number of short grooming	1,6±0,4	1,2±0,4	0,30
Number of long grooming	2,6±0,5	2,9±0,6	0,90
Number of contacts with "familiar" object	2,6±0,7	1,9±0,4	0,58
Number of contacts with "new' object	2,9±0,6	2,9±0,5	0,90
Number of active contacts with "familiar" object	1,2±0,6	0,1±0,1	0,25

Number of active contacts with "new" object	1,8±0,4	0,1±0,1	0,002
Number of freezing episodes	16,8±2,1	23,2±2,2	0,07
Total freezing time, sec	175,4±46,4	236,7±38,6	0,14
Stereotypy (head waiving)	1,9±0,7	0,9±0,4	0,30

Statistical significance is calculated according to Mann-Whitney U-test. Significantly differing parameters are underlined. Data are represented as the mean ± SEM

Table S7A. Social dominance "tube" test, between groups comparisons. Number of wins, for KM and Wistar groups

	Wins, N	Loses, N	Dead heat, N
KM rats	20	12	8
Wistar rats	12	20	8

KM rats won with a higher probability. Chi-squared x2=4, 00; p<0,05.

Table S7B. Behavioral parameters registered in "the social dominance in a tube" test, between groups comparisons.

	KM rats, (mean±sem)	Wistar rats (mean±sem)	p-value
Win latency	111,6±14,7	39,2±19	0,002
Number of pushes	9,4±1,5	13,5±1,5	0,02
Number of forward propulsions	2,3±0,4	2,1±0,4	0,71
Number of retreats	1,0±0,4	3,6±0,4	0,001

Statistical significance is calculated according to Mann-Whitney U-test. Significantly differing parameters are underlined. Data are represented as the mean ± SEM
